# A comparison of opioid-containing anesthesia versus opioid-free anesthesia using the Cortínez-Sepúlveda model on differential cytokine responses in obese patients undergoing gastric bypass surgery: a randomized controlled trial

**DOI:** 10.1186/s12871-022-01838-8

**Published:** 2022-09-16

**Authors:** Wendy Campos-Pérez, Lilia Ramírez-Plascencia, Mariana Pérez-Robles, Juan J. Rivera-Valdés, Patricia Sánchez-Muñoz, Liliana Pérez-Vargas, Dulce González-Landeros, Juan Heberto Muñoz Cuevas, Erika Martínez-López

**Affiliations:** 1 Institute of Translational Nutrigenetics and Nutrigenomics, Department of Molecular Biology and Genomics, University Center of Health Sciences, University of Guadalajara, Sierra Mojada 950, 44340 Guadalajara, Jalisco Mexico; 2 Anesthesiology Service, Civil Hospital of Guadalajara “Juan I Menchaca”, Salvador Quevedo y Zubieta 750, 44340 Guadalajara, Jalisco Mexico; 3 Bariatric and Metabolic Surgery Service, Civil Hospital of Guadalajara “Juan I Menchaca”, Salvador Quevedo y Zubieta 750, 44340 Guadalajara, Jalisco Mexico

**Keywords:** Opioid free anaesthesia, Fentanyl, Cortínez-Sepúlveda model, IL-6

## Abstract

**Background:**

Opioid anesthetic agents can modulate the impaired immune response in obese patients through mechanisms that involve the expression and release of cytokines. For this reason, anesthetic care for obese patients remains controversial. Therefore, the aim of the study was to compare the effect of opioid-containing anesthesia (OCA) vs opioid-free anesthesia (OFA) using the Cortínez-Sepúlveda model on IL-6, IL-1β and TNF-α serum levels before and after surgery in obese patients undergoing bypass surgery.

**Methods:**

This randomized cross-sectional study conducted among 40 unrelated obese adults was performed in the Civil Hospital of Guadalajara “Dr. Juan I. Menchaca”. Before undergoing laparoscopic Roux-en-Y gastric bypass, patients were randomly assigned to two anesthesia groups: OCA (*n* = 20) or OFA (*n* = 20). Fentanyl was the opioid used in the OCA group. The Cortínez-Sepúlveda pharmacokinetic model was used to characterize the disposition of intravenous propofol for the target-controlled infusion technique in obese patients. Body mass was determined to the nearest 0.05 kg using a balance scale (Seca 703; Seca, Hamburg, Germany). Blood samples were taken before and immediately after surgery and cytokine concentrations were determined by ELISA. Pain was assessed using a numerical pain rating scale. Adverse effects were collected within the first 24 h after surgery.

**Results:**

A total of 6 men and 34 women were included (37.9 ± 10.6 years). Pre-surgery IL-6 and TNF-α serum levels were not detected in study subjects. However, IL-1β levels significantly decreased after surgery (49.58 pg/mL (18.50–112.20)-before surgery vs 13 pg/mL (5.43–22)-after surgery, *p* = 0.019). IL-6 concentrations were significantly higher in subjects who received OCA (with fentanyl) compared to subjects with OFA (224.5 pg/mL (186.3–262.8) vs 99.5 pg/mL (60.8–138.2), respectively, *p* < 0.001; adjusted by age, gender, and BMI). In addition, the use of opioids confers an increased risk for higher IL-6 levels in obese patients (OR = 2.95, 95% CI: 1.2–7.2, *p* = 0.010). A linear regression model showed that the operative time (in hours) of bypass surgery and anesthetic technique were positively correlated with IL-6 levels.

**Conclusion:**

Anesthesia with opioids correlated positively with IL-6 serum levels in obese patients undergoing bypass surgery. This finding could have clinical relevance when an appropriate anesthetic management plan is selected for bariatric surgical patients.

**Trial registration:**

The study was retrospectively registered at ClinicalTrials.gov Identification Number: NCT04854252, date 22/04/2021.

## Background

Obesity is a multifactorial disease caused by an imbalance between daily energy intake and energy expenditure. It has been associated with low-grade chronic inflammation and comorbidities, such as type 2 diabetes mellitus, cardiovascular diseases, and hyperlipidemia [1]. Furthermore, obesity is a significant public health problem that affects 40.2% of women and 30.5% of men in Mexico [2], and over 500 million adults worldwide [1].

Weight loss surgery has been shown to be an effective treatment for obesity and secondary comorbidities [3]. However, the interindividual variability of adipose tissue distribution and the presence of comorbidities, increase the risk of surgical complications in obese patients. Therefore, perioperative care and the type of anesthesia represent a great challenge for these patients [4]. Although efforts have been made to develop standardized guidelines or protocols for anesthetic care in patients with obesity [5], an ideal anesthesia technique or drug combination are still unknown [4].

Total intravenous anesthesia (TIVA) is a technique where anesthesia is exclusively administered intravenously or in combination with medication in the absence of any inhaled anesthetic agents [6]. The main drugs used in TIVA are thiopental, morphine, fentanyl, ketamine, dexmetomidine, propofol, and midazolam; fentanyl and morphine are synthetic and natural opioids, respectively. On the other hand, opioid-free anesthesia (OFA) is a technique in which no intraoperative systemic, neuraxial, or intracavitary opioid is administered. The prevention and risk reduction associated with the use of opioids such as respiratory depression, central muscle rigidity, pharyngeal muscle weakness, negative inotropism, nausea, vomiting, constipation, urinary retention, drowsiness, and hyperalgesia, among others, supports the use of OFA in patients undergoing bariatric surgery [7].

Since obesity produces low-grade chronic inflammation, which is also associated with an increase of circulating pro-inflammatory factors, it has been reported that anesthetic agents can modulate the impaired immune response in patients with obesity [4]. The opioid-mediated immune activity can have immunosuppressive, immunostimulant, or dual effects [8]. The mechanisms involved include direct action on inflammatory cells by altering cytokine release [9], cytokine receptor expression [10], phagocytosis or cytotoxic actions [11], and transcription or translation of protein mediators [12]. Hence, there is a need for further research on the immune response of patients exposed to different anesthetic drugs. Therefore, the aim of this study was to compare the effect of opioid-containing anesthesia (OCA) vs OFA on IL-6, IL-1β and TNF-α serum levels before and after surgery in patients with obesity undergoing bypass surgery using the Cortínez-Sepúlveda model.

## Methods

The study was approved by the Ethics Committee for Human Research of the Civil Hospital of Guadalajara “Dr. Juan I Menchaca” (Registration number: 17CI14 039116 COFEPRIS). Written informed consent was obtained from all subjects after they were informed about the research procedures. Besides, the study was conducted in accordance with the Declaration of Helsinki (2013) guidelines. The trial was retrospectively registered at clinicaltrial.gov (NCT04854252, Martínez-López E, PhD, 22/04/2021). This manuscript adheres to the applicable Enhancing the QUAlity and Transparency of Health Research (EQUATOR) guidelines.

### Sample size and randomization

The sample size was determined using the formula for estimating the difference in effect with preliminary data obtained in our hospital and previously reported in the literature [13] and in accordance with the following assumptions: α of 0.05 (two-tailed), power of 90%, Zα of 1.96 (considering a normal distribution at a confidence level of 95), and Zβ of1.282 (value equivalent to β = 0.10 with a power of 90%). Therefore, a minimum of 15 patients per group was calculated, however, a formula for the correction for losses or abandonments was used, then a minimum of 19 patients per group was considered. Patients who met the inclusion criteria were recruited, and a computer-generated list of random numbers was used for identification of participants; then, patients were randomized 1:1 by the anesthesiologist and scheduled for their bypass surgeries until the sample size in each group was completed.

### Study subjects

In this randomized cross-sectional study, a total of 40 unrelated adults aged 18–65 years from western Mexico who were scheduled for bypass surgery at the Service of Anesthesiology and Bariatric Surgery of the Civil Hospital of Guadalajara “Dr. Juan I Menchaca”, Jalisco, Mexico were recruited. The past medical history of study subjects was assessed. The inclusion criteria were patients aged 18 to 60 years, with a BMI > 30 kg/m^2^ who were scheduled to undergo a gastric bypass after an integrated preoperative evaluation and signed the informed consent. The exclusion criteria were: patients with a history of ischemic heart disease, history of drug abuse, and with any known allergy to any of the drugs used during anesthesia. Elimination criteria were: patients who withdrew their consent or with insufficient and poor quality blood samples (coagulated) or other reasons that did not allow sample processing.

### Anthropometric variables

Anthropometric parameters were measured after an 8–10 hour overnight fast. Measurements were performed with light clothes and without shoes. Height measurement was determined using a stadiometer with a precision of 1 mm (Rochester Clinical Research, Inc., Rochester, NY, USA). Body mass was determined to the nearest 0.05 kg using a balance scale (Seca 703; Seca, Hamburg, Germany) with subjects in underwear. Body mass index (BMI) was calculated as kg/m^2^ and interpreted according to WHO specifications where subjects with a BMI of 30–34.9 kg/m^2^ were considered as type I obesity, a BMI of 35–39.9 kg/m^2^ as type II obesity, and ≥ 40 kg/m^2^ as obesity type III [14].

### Anesthesia management

#### TIVA opioid-containing anesthesia

Before the laparoscopic Roux-en-Y gastric bypass (LRYGB) as a bariatric procedure, subjects were randomly assigned to the anesthesia study groups with (OCA) or without an opioid (OFA). The general anesthesia technique was defined as a combination of medications administered intravenously in the absence of any inhaled anesthetic agent. Patients undergoing OCA received the following medication schedule:

Loading dose: fentanyl in a bolus dose of 3 mcg/kg (corrected weight), propofol 2–2.5 Cp by target-controlled infusion (TCI) with the Cortínez-Sepúlveda pharmacokinetic model (real weight) [15], ketamine 0.15 mg/kg (corrected weight), lidocaine 2% 1 mg/kg (corrected weight), magnesium sulfate 30–50 mg / kg (corrected weight), and rocuronium bromide in a bolus dose of 0.6 mg/kg (real weight).

Maintenance dose: fentanyl 0.003–0.006 mcg/kg/minute (corrected weight), propofol 2–2.5 Cp with the Cortínez-Sepúlveda pharmacokinetic model (real weight) [15], ketamine 0.15 mg/kg/ minute (corrected weight), lidocaine 2% 1 mg/kg (corrected weight), magnesium sulfate 10 mg/kg/minute (corrected weight), and rocuronium bromide 1.25 mcg/ Kg/minute (corrected weight).

#### TIVA opioid-free anesthesia

TIVA opioid-free anesthesia technique is defined as a combination of medications administered intravenously in which no intraoperative systemic, neuraxial or intracavitary opioid is administered during anesthesia and it also avoids opioids in the perioperative period. Patients undergoing OFA received the following medication schedule:

Loading dose: dexmedetomidine 1–1.5 mcg/kg (corrected weight) for 40 minutes, propofol 2.5–3.5 Cp with the Cortínez-Sepúlveda pharmacokinetic model (real weight) [15], ketamine 0.15 mg/kg (corrected weight), lidocaine 2% 1 mg/kg (corrected weight), magnesium sulfate 30–50 mg/kg (corrected weight), and rocuronium bromide in a bolus dose of 0.6 mg/kg (real weight).

Maintenance dose: dexmedetomidine 0.3–0.7 mcg/kg/minute (corrected weight), propofol 2–4 Cp with the Cortínez-Sepúlveda pharmacokinetic model (real weight), ketamine 0.15 mg/kg/ minute (corrected weight), lidocaine 2% 1 mg/kg (corrected weight), magnesium sulfate 10 mg/kg/minute (corrected weight), and rocuronium bromide 1.25 mcg/kg/minute (corrected weight).

All patients were reverted from deep rocuronium-induced neuromuscular blockade with sugammadex at a dose of 2–4 mg / kg (corrected weight) in train-of-four (TOF) monitoring.

Besides, TCI calculated with the Cortínez-Sepúlveda pharmacokinetic model was performed with Syramed® SP6000 Premium Syringe Pump (Arcomed AG, Switzerland).

### Depth of anesthesia monitoring

To assess the depth of anesthesia and state of consciousness, indicators of hypoxia and brain death, the Spectral Edge Frequency (SEF) of processed real-time electroencephalography (EEG) was used during surgery.

### Postoperative analgesia technique

Continuous infusion at the post anesthesia care unit the following protocols were used for 24 hours:

- OCA group: metamizole 30 mg/kg (corrected weight), ketamine 0.5 mg/kg (corrected weight), magnesium sulfate 5 mg/kg (corrected weight), lidocaine 1 mg/kg (corrected weight), buprenorphine 1 mcg/kg (corrected weight), plus paracetamol 1 g/ kg (corrected weight) every 12 hours.
- OFA group: metamizole 30 mg/kg (corrected weight), ketamine 0.5 mg/kg (corrected weight), magnesium sulfate 5 mg/kg (corrected weight), lidocaine 1 mg/kg (corrected weight), plus paracetamol 1 g/ kg (corrected weight) every 12 hours.

### Primary and secondary outcomes

Basal and post-surgery cytokine serum levels IL-1β, IL-6, and TNF-α were considered as the primary outcome in this study, while secondary outcomes were the post-surgery pain estimation and post-surgery adverse effects.

### Cytokine measurements

Blood samples were taken before and immediately after surgery in a Vacutainer® tube and then centrifuged for 15 minutes at 3500 rpm at 4 °C to obtain the serum. Serum samples were stored at − 80 °C until analysis. Cytokine serum levels were measured with an enzyme-linked immunosorbent ***(***ELISA) assay. LEGEND MAX™ Human IL-1β (cat # 437007), LEGEND MAX™ Human IL-6 (cat # 430507) and LEGEND MAX™ Human TNF-α (cat # 430207) ELISA kits were used according to the supplier’s instructions.

### Pain assessment

After 24 h of surgery and using a numerical pain rating scale (NPRS), patients were asked to circle the number between 0 and 10 that best fits their pain intensity where 0 represented “no pain at all” and 10 “the worst pain ever” [16]. Therefore, there were 11 possible answers between 0 and 10.

### Adverse effects

Regarding adverse effects, nausea and vomiting were reported within the first 24 h after surgery.

### Statistical analysis

The Shapiro Wilk test was used to analyze normal distribution of quantitative variables. Crude means or medians between groups were compared using Student’s t-test or a Mann-Whitney U test, respectively. The general univariate linear model adjusted for other quantitative variables was used to compare differences between the two study groups. Moreover, the chi-square test was used to compare two categorical variables. To determine if the presence of one of the variables behaves as a risk factor for higher cytokine levels, an odds ratio (OR) test was performed. Regarding the evaluation of the individual and joint effect of two or more factors (anesthetic technique, hours of surgery and BMI) on a quantitative dependent variable, a linear regression model was carried out. The analysis plan was developed before accessing the data.

Statistical analyses were performed using the Statistical Package for the Social Sciences (SPSS), version 20.0 software (IBM Corp., Armonk, NY, USA). A confidence interval of 95% was set and a *p* < 0.05 was considered statistically significant.

## Results

### Population description

**Fig. 1:**
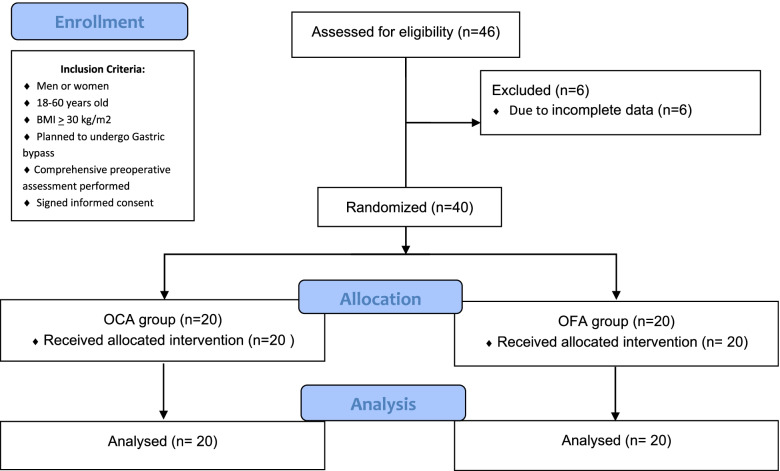
Flowchart of study subjects. BMI: Body Mass Index, OCA: Opiod-containing anesthesia, OFA: Opiod-free anesthesia

Forty subjects (six men and 34 women with a mean age of 37.9 ± 10.6 years) were recruited in this study from November 2020 to March 2021 (Fig. 1). Fifty percent (*n* = 20) of subjects received OFA, while the other half received OCA, specifically fentanyl. The Spectral Edge Frequency (SEF) values ranged between 15 and 10 (data not shown), so there was no data of suppression or brain death in any of the patients during surgery. In turn, no cases of infection, mortality, readmission, or the need for additional surgery occurred in any of the study groups. Since hemodynamic instability was not present during the trans operative period, a regional anesthesia technique was not required. Patient discharge from the post anesthesia care unit occurred after 24 hours of the patients’ arrival.

Regarding cytokines, it should be noted that pre-surgery serum IL-6 levels were not detected. On the other hand, when comparing the concentrations of IL-1β in pre-surgery and post-surgery subjects, significant differences were found (49.58 pg/mL (18.50–112.20) vs 13 pg/mL (5.43–22) respectively, *p* = 0.019). Other descriptive variables are shown in Table 1. TNF-α concentrations were also not detected before or after surgery in all study subjects.

**Table 1:** Characteristics of study subjects

Variables	(***n*** = 40)
BMI (kg/m^2^)	43.7 ± 6.2
OB I n (%)	11 (27.5%)
OB II n (%)	29 (72.5%)
IL-6 pre-surgery (pg/mL)	ND
IL-6 post-surgery (pg/mL)	162.7 ± 106.5
IL-β pre-surgery (pg/mL)	49.58 (18.50–112.20)^a^
IL-1β post-surgery (pg/mL)	13 (5.43–22)^a^
TNF-α pre-surgery (pg/mL)	ND
TNF-α post-surgery (pg/mL)	ND
NPRS	0 (0–3)^a^
Nausea, n (%)	8 (20%)
Vomiting, n (%)	3 (7.5%)

*BMI* body mass index, *IL-6* interleukin 6, *IL-1β* interleukin 1β, *NPRS* Numerical Pain Rating Scale, *ND* not detected, *TNF-α* Tumor necrosis factor alpha

^a^Data expressed in median and interquartile range

### Characteristics of study subjects by anesthetic technique

When comparing the variables of subjects classified by anesthetic technique, IL-6 concentrations were significantly higher in subjects who received OCA compared to subjects with OFA (224.5 (186.3–262.8) pg/mL vs 99.5 (60.8–138.2) pg/mL, respectively, *p* < 0.001; adjusted by age, gender, and BMI). In addition, patients who received OCA were 2.9 times more likely to have higher levels of IL-6 (OR = 2.95, 95% CI: 1.2–7.2, *p* = 0.010). The other variables are shown in Table 2.

**Table 2:** Characteristics of study subjects by anesthetic technique

Variables	Opioid-containing anesthesia (OCA) (***n*** = 20)	Opiod-free anesthesia (OFA) (***n*** = 20)	***P*** value
Age (years)	37 ± 10.3	38.8 ± 11.1	0.6^׀^
Women (n)	15	19	0.18*
Men (n)	5	1	
BMI (kg/m^2^)	45 (42.1–47.9)	42.4 (39.5–45.3)	0.211 ^׀^
OB I (n) %	1 (5%)	10 (50%)	0.003*
OB II (n) %	19 (95%)	10 (50%)	
IL-6 pre-surgery (pg/mL)	ND	ND	**–**
IL-6 post-surgery (pg/mL)	224.5 (186.3–262.8)	99.5 (60.8–138.2)	**< 0.001**^**׀**^
IL-1β pre-surgery (pg/mL)	101.2 (51.9–254.3)	231.6 (48.3–414.9)	0.281 ^**׀**^
IL-1β post-surgery (pg/mL)	15.7 (3.3–28)	14.86 (4.5–25.2)	0.921 ^**׀**^
TNF-α pre and post-surgery (pg/mL)	ND	ND	–
NPRS	1.4 (0.4–2.4)	1.7 (0.7–2.7)	0.665 ^**׀**^
Nausea, n (%)	6 (30%)	2 (10%)	0.2*
Vomiting, n (%)	3 (15%)	0 (0%)	0.2*

*BMI* body mass index, *IL-6* interleukin 6, *IL-1β* interleukin 1 beta, *NPRS* Numerical Pain Rating Scale, *ND* not detected, *TNF-α* Tumor necrosis factor alpha

* Chi square test, ׀ Values are shown as estimated means and the confidence interval, adjusted by age, gender, and BMI

### Association between operative time (hours of surgery) and anesthetic technique with serum IL-6 levels

Due to IL-6 serum concentrations were statistically different between anesthetic techniques, the relation of hours of surgery and BMI with serum IL-6 by a linear regression model was also performed to know if other variables were related and, it was demonstrated that more operative time (hours of surgery) and OCA were positively correlated with IL-6 levels (Table 3) but not the BMI (*p* = 0.165).

**Table 3:** Linear regression model of factors associated with serum IL-6 levels

IL-6 (pg/mL)	B Coefficient (95% CI)	R^**2**^	***P*** value
**Operative time (hours of surgery)**	54 (10.3–98.6)	0.027	0.017
**Anesthetic technique: OCA**	81.6 (18.5–144)	0.042	0.013

*OCA* Opiod-containing anesthesia

## Discussion

Obesity and intravenous anesthetics modulate the immune response through indirect effectors of immunity, such as cytokines [17]. Among the cytokines that are considered the most relevant biomarkers of the inflammatory response during surgical procedures are TNF-α, interleukin IL-6, and IL-1β [18, 19].

Besides, it is well known that morbid obesity is related to low-grade and chronic systemic inflammation and immune activation, however in this study, serum TNF-α concentrations were not detected before (fasting state) or after the surgery. This finding could be related to the fact that proinflammatory cytokines like TNF-α, TGF-β, IL-1, IL-6, IL-8, IL-10, IFN-γ, haptoglobin, complement factors, chemoattractant cytokines, and some hormones (like leptin) are synthesized and released in human adipose tissue [20]. Therefore, future studies should consider measuring cytokines as well as their expression in adipose tissue to elucidate the mechanisms involved in the immune response of obese population.

Nevertheless, a study where fasting serum TNF-α was measured by an ELISA assay in 15 patients with android obesity, 13 patients with gynoid obesity and 15 lean healthy controls with normal glucose tolerance and blood pressure reported higher TNF-α concentrations in subjects with android obesity (8.92 ± 0.44 pg/mL, *p* < 0.01 vs controls) compared with gynoid obesity (7.01 ± 0.30 pg/mL) and lean controls (6.88 ± 0.26 pg/mL). Authors concluded that TNF-α may be one of the factors that contributes to vascular dysfunction in subjects with android obesity [21].

Regarding IL-6, it is considered one of the most important pro-inflammatory interleukins produced by monocytes and macrophages. IL-6 is critical in the development of the acute phase response during inflammation [22]. In addition to its role in the inflammatory response, IL-6 is vital in host defense, immune responses, wound healing, and hematopoiesis [23, 24]. Serum IL-6 levels have been shown to change during surgical procedures (in response to trauma) as well as during anesthesia. In this study, serum IL-6 levels at the preoperative level could not be detected in study subjects, however, after surgery, serum levels were higher in patients with opioid anesthesia, which is in accordance with findings by Høgevold HE, et al. where serum IL-6 levels increased rapidly after total hip replacement surgery, concluding that IL-6 levels are indicators of the extent of tissue damage [25].

In the present study a positive association between serum IL-6 concentration and TIVA opioid-containing anesthesia was found. In this sense, Yong-Min Liu et al., evaluated the effects of tramadol on the proinflammatory responses in a rat model and they found that serum IL-6 levels increased 2 hours after the administration of anesthesia (40 ± 5 vs 100 ± 5 pg/ml, *p* < 0.01) [26]. Therefore, they suggested that IL-6 serum levels could be a useful marker of tissue damage because it has been reported that IL-6 correlates with surgery time, trauma degree, and clinical post-surgery complications [27, 28]. Moreover, in a study conducted in 16 women undergoing abdominal hysterectomy, IL-6 concentrations increased significantly in serum 2 hours after administering 15 mg/kg of fentanyl [3.5 (3.5–3.7) vs 40.3 (17.7–45.7) pg/mL *p* < 0.01] [29]. This study also found that the operative time (hours of surgery) is decisive for serum concentrations of IL-6. Serum levels of this cytokine have shown a positive correlation with the time of surgery (*r* = 0.554, *p* < 0.01) and the amount of blood lost during surgery (*r* = 0.427, *p* < 0.01) since IL-6 is an important mediator in response to trauma [16]. Previous studies have shown the clinical relevance of the IL-6 and other interleukins, since they can be useful as predictive factors that can identify patients at risk for postoperative complications [30]. Furthermore, Il-6 has been described as a good predictor of morbidity and mortality [31], cardiac complications and shorter duration of mechanical ventilation [32].

Regarding pre-surgery and post-surgery IL-1β serum concentrations in all study subjects, significant differences were found since values obtained after surgery were lower for both study groups (*p* = 0.029). This differs from clinical studies where IL-1β concentrations did not change during and after the conclusion of abdominal surgery [33]. Nonetheless, an increase in IL-1β concentration has been reported 48 hours after surgery when using ketamine as an anesthetic [13]. IL-1β is expressed in macrophages, NK cells, monocytes, and neutrophils [34] and is a potent pro-inflammatory cytokine capable of inducing an adaptive cellular response in Th1 and Th17 lymphocytes. It has been also associated with exacerbation of damage in chronic diseases and tissue injuries [35].

Serum cytokine levels could be therapeutically relevant in surgical interventions. The systemic inflammatory response during and after a surgical procedure is considered an important factor for the development of postoperative complications, for instance, increased susceptibility to infection, inappropriate reactions to stress, and hypercatabolism [36, 37]. The adjustment of the immune response related to anesthesia could help reduce perioperative morbidity through the reduction of pro-inflammatory cytokines [38].

It is known that opioids have a bidirectional influence on most aspects of the immune response associated with leukocytes, macrophages, mast cells, lymphocytes, and NK cells [39]. Therefore, the administration of OFA is emerging as a better anesthetic management strategy for acute pain with other groups of drugs that are assumed to have a comparable analgesic effect without affecting the immune system, such as dexmedetomidine, especially in patients with impaired immune response like patients with obesity, opioid addiction, or with an oncological condition [7, 40, 41].

It has been reported that dexmedetomidine inhibits the maturation and activity of dendritic cells and limits the proliferation of helper lymphocytes and cytotoxic activity through activation of the alpha-2-adrenergic receptors (α2) [42, 43], thus changing the activity of potassium and calcium channels in the cell membrane of spinal cord neurons with analgesic, hypnotic, and sedative effects. Besides, dexmedetomidine has been recognized for presenting anti-inflammatory effects, by reducing postoperative levels of IL-6, IL-8, and TNF- α, although the evidence is still diverse and heterogeneous [44].

However, it is important to note that among the contraindications in OFA use are circulatory insufficiency, cardiac arrhythmias (especially bradyarrhythmias), hypovolemia, shock, unstable coronary artery disease, autonomic neuropathy with orthostatic hypotension, and history of allergic reactions [7, 40]. Therefore, the selection of this therapy must be individualized considering patient comorbidities.

Moreover, limitations of this study include the small sample size and the absence of tissue cytokine concentration measurements. Despite not detecting pre-surgical IL-6 values, this study highlights the differences of the inflammatory response when using OCA or OFA in patients undergoing gastric bypass, specifically, the positive association between OCA and higher serum IL-6 concentrations. Therefore, further studies that measure cytokine levels and expression at various intervals before and after surgery in different tissues are needed to address the clinical significance of serum cytokine levels as markers of inflammation in the anesthetic management of patients with obesity.

Finally, it is important to address that the Cortínez-Sepúlveda model was used because it has been recently shown that it derived from the need to avoid inadequate intravenous anesthetics in obese patients with higher and anticipated plasma propofol concentrations. Cortínez et al. derived and validated a propofol model to perform effect-site TCI in obese patients, concluding that this model derived exclusively from patients with obesity data and is not recommended for TCI in lean patients due to the risk of underdosing [15, 45].

## Conclusion

In conclusion, our results demonstrated that OCA correlated positively with serum IL-6 levels in patients undergoing bypass surgery. IL-6 serum levels may be an indicator of inflammatory responses and it could be clinically relevant when choosing an appropriate anesthetic management for obese patients who undergo bypass surgery.

## Data Availability

The data that support the findings of this study are available from the corresponding author, but restrictions apply to the availability of these data, which were used under license for the current study, and so are not publicly available.

## Abbreviations

BMI: Body mass index
EEG: Electroencephalography
EQUATOR: Enhancing the quality and Transparency of Health Research
LRYGB: Laparoscopic Roux-en-Y gastric bypass
NPRS: Numerical pain rating scale
OCA: Opioid-containing anesthesia
OFA: Opioid-free anesthesia
OR: Odds ratio
SEF: Spectral Edge Frequency
SPSS: Statistical Package for the Social Sciences
TCI: Target-controlled infusion
TIVA: Total intravenous anesthesia
TOF: Train-of-four
IL-6: Interleukin 6
IL-1β: Interleukin 1β
TNF-α: Tumor necrosis factor alpha
ND: Not detected
